# Efficacy of transarterial chemoembolization with drug-eluting beads combined with systemic chemotherapy and targeted therapy in colorectal cancer liver metastasis

**DOI:** 10.1186/s12957-023-03253-w

**Published:** 2023-12-01

**Authors:** Yen-Cheng Chen, Ching-Wen Huang, Ching-Chun Li, Tsung-Kun Chang, Wei-Chih Su, Po-Jung Chen, Yung-Sung Yeh, Yu-Tang Chang, Hsiang-Lin Tsai, Ming-Chen Paul Shih, Jaw-Yuan Wang

**Affiliations:** 1 Graduate Institute of Clinical Medicine, College of Medicine, Kaohsiung Medical University, Kaohsiung, Taiwan; 2 Division of Colorectal Surgery, Department of Surgery, Kaohsiung Medical University Hospital, Kaohsiung Medical University, No. 100 Tzyou 1St Road, Kaohsiung, 807 Taiwan; 3 Department of Surgery, Faculty of Medicine, College of Medicine, Kaohsiung Medical University, Kaohsiung, Taiwan; 4 Department of Surgery, Faculty of Post-Baccalaureate Medicine, College of Medicine, Kaohsiung Medical University, Kaohsiung, 80708 Taiwan; 5 Division of Trauma and Surgical Critical Care, Department of Surgery, Kaohsiung Medical University Hospital, Kaohsiung Medical University, Kaohsiung, 80708 Taiwan; 6 Department of Emergency Medicine, Faculty of Post-Baccalaureate Medicine, College of Medicine, Kaohsiung Medical University, Kaohsiung, 80708 Taiwan; 7 Graduate Institute of Injury Prevention and Control, College of Public Health, Taipei Medical University, Taipei, 11031 Taiwan; 8 Division of Pediatric Surgery, Department of Surgery, Kaohsiung Medical University Hospital, Kaohsiung Medical University, Kaohsiung, 80708 Taiwan; 9 Department of Medical Imaging, Kaohsiung Medical University Hospital, Kaohsiung Medical University, Kaohsiung, 80708 Taiwan; 10 Graduate Institute of Medicine, College of Medicine, Kaohsiung Medical University, Kaohsiung, 80708 Taiwan; 11 Center for Cancer Research, Kaohsiung Medical University, Kaohsiung, 80708 Taiwan

**Keywords:** Colorectal cancer with liver metastasis, Drug-eluting bead, Trans-arterial chemoembolization, Targeted therapy, Chemotherapy

## Abstract

**Background:**

Systemic therapy is the standard treatment for unresectable colorectal cancer with liver metastasis (CRCLM). Transarterial chemoembolization with drug-eluting beads (DEB-TACE) is considered an effective treatment option for CRCLM. Few studies have investigated the combination of DEB-TACE, chemotherapy, and targeted therapy for CRCLM. In the present study, we evaluated the disease control rate (DCR), adverse events, and survival among patients with CRCLM who underwent the combination of DEB-TACE and chemotherapy/targeted therapy.

**Materials:**

We retrospectively reviewed 35 patients with CRCLM who were treated between January 2015 and January 2021. Standard systemic chemotherapy, targeted therapy, and 66 DEB-TACE procedures were administered. Data were collected on each DEB-TACE procedure, including chemotherapy agents, tumor burden of liver metastasis, number of DEB-TACE courses, and adverse events. Patients who received DEB-TACE after failure of first-line systemic therapy were categorized into the first-line failure group. Patients who received DEB-TACE after the failure of second-line, third-line, or fourth-line therapy were categorized into the other group. Subgroup analysis was performed to compare overall survival (OS) and progression-free survival (PFS) between the two groups.

**Results:**

In total, 35 patients with CRCLM (34 patients with adenocarcinoma and 1 patient with neuroendocrine carcinoma) were enrolled. In total, 13 patients (37.1%) had extrahepatic metastases at initial diagnosis. In this study, 66 DEB-TACE procedures were performed. The DCR was 54.3%. The median OS period was 47.4 months, and the estimated 3-year OS rate was 59.5%. The median PFS period was 6.3 months, and the estimated 1-year PFS rate was 20.6%. The PFS period was longer in the first-line failure group than in the other group (7.2 vs. 6.3 months). No significant difference was observed in OS between the two groups. Four episodes (6.1%) of grade 3 intra-abdominal infection were observed.

**Conclusion:**

The combination of chemotherapy, targeted therapy, and DEB-TACE can lead to a favorable DCR and survival outcomes in patients with CRCLM. Early intervention with DEB-TACE (i.e., after the failure of first-line therapy) has the potential to extend the PFS period in patients with CRCLM. Severe adverse events were rare and manageable. Further prospective, randomized controlled studies are warranted to obtain more conclusive findings.

## Background

Colorectal cancer (CRC) is the third most common malignant disease worldwide and is a global public health concern. Treatment of metastatic CRC (mCRC) is clinically challenging [1, 2]. Studies have estimated that 20–25% of patients with stage IV CRC have synchronous distant metastasis [3, 4]. Approximately 30% of patients who underwent primary resection for CRC developed metachronous metastasis [5]. CRC most commonly metastasizes to the liver, followed by the lung and peritoneum [6]. The initial treatment for CRC with liver metastasis (CRCLM) is systemic chemotherapy with 5-fluorouracil, oxaliplatin, fluoropyridine, and irinotecan [7, 8]. Adjuvant targeted therapy, such as bevacizumab or cetuximab, can provide additional survival benefits [6, 7, 9]. Surgical resection (i.e., metastasectomy) after systemic therapy provides the best prognosis [3, 6]. However, 70–75% of patients with CRCLM cannot tolerate liver resection surgery, or their liver metastasis remains unresectable after systematic therapy [10, 11]. Additional local therapies, including transarterial chemoembolization (TACE), ethanol injection, radiofrequency ablation, cryotherapy, and microwave ablation, are used to control liver metastasis [3, 12].

TACE is commonly performed for unresectable hepatocellular carcinoma. TACE exerts its therapeutic effects through cytotoxicity and ischemia as the primary mechanisms [13]. Embolization-induced ischemia is insufficient to control CRCLM due to the characteristics of hypovascular tumors [14]. However, the major blood supply system of metastatic tissue is hepatic arterial circulation. Therefore, transarterial chemotherapy can be effective and cause minimal damage to normal liver tissue [14]. The therapeutic effect of transarterial chemotherapy can be strengthened with the use of TACE with drug-eluting beads (DEB-TACE) [15]. Drug-eluting beads (DEBs) can provide the continuous intra-arterial release of chemotherapy agents, such as irinotecan and epirubicin [11, 12]. Studies have demonstrated that DEB-TACE can safely and effectively control CRCLM [10, 16] and that it provides acceptable tumor responses and survival benefits [3, 8, 17].

Nevertheless, the role of TACE in CRCLM treatment is still being explored. TACE has been proven to be effective as a palliative therapy for CRCLM [3, 10]. With advancements in chemotherapy agents and DEBs, TACE can play an important role as a neoadjuvant or symptomatic therapy [17]. The combination of targeted therapy and chemotherapy is the standard treatment for stage IV CRC [18]. Whether TACE intervention combined with standard systemic therapy, especially with targeted therapy improves outcomes in patients with CRCLM remains unknown. In the present study, we retrospectively evaluated 35 patients with CRCLM who received DEB-TACE with a standard treatment course of targeted therapy and chemotherapy. The real-world data, including disease control rate (DCR), adverse events, and patient survival, were analyzed.

## Materials and methods

### Patients

#### Inclusion and exclusion criteria

From January 2015 to January 2021, a total of 35 patients with CRCLM who received DEB-TACE with a standard treatment course of targeted therapy and chemotherapy were enrolled retrospectively. Patients were excluded if they were aged < 18 years; if they had a history of synchronous malignancies other than non-melanoma skin cancer, bleeding tendency, unstable vital signs, severe liver function impairment, severe cardiovascular comorbidities, or major medical comorbidities that may affect treatment compliance; or if they were pregnant. Data on DEB-TACE details, patient characteristics, biochemistry examinations, image features, adverse events, systemic therapy agents, and oncologic outcomes were collected from medical records. The present study was approved by the institutional ethics committee of our hospital (KMUHIRB-E(II)-20,220,041).

#### Systemic therapy

The mutation status of the *RAS* and *BRAF* genes was determined before targeted therapy was applied. Polymorphism of uridine diphosphate glucuronosyltransferase 1A1 was surveyed for guidance on irinotecan dose escalation [19]. The CRC evaluation and treatment protocols were conducted following the principles described in our previous study [20]. Diagnoses were confirmed by abdominal computed tomography (CT), magnetic resonance imaging (MRI), colonoscopy, and histopathologic findings. Tumor-sidedness was distinguished based on colon splenic flexure. A multidisciplinary team including a colorectal surgeon, gastroenterologists, medical oncologists, radiologists, radiation oncologists, and pathologists discussed treatment programs. All patients were given diagnoses of unresectable CRCLM. Neoadjuvant systemic therapy including targeted therapy and chemotherapy was applied for all patients in accordance with the National Comprehensive Cancer Network guidelines and the Taiwan Society of Colon and Rectal Surgeons Consensus on mCRC Treatment [18, 21]. For patients with adenocarcinoma, the first-line chemotherapy regimen was FOLFIRI (folinic acid, 5-fluorouracil, and irinotecan) or FOLFOXIRI (folinic acid, 5-fluorouracil, oxaliplatin, and irinotecan). An anti-vascular endothelial growth factor agent (bevacizumab; Avastin; Roche, Basel, Switzerland) or an anti-epidermal growth factor receptor agent (cetuximab; Erbitux; Merck, Darmstadt, Germany; or panitumumab, Vectibix; Amgen, CA, USA) was chosen as the first-line treatment agent depending on the patient’s *RAS* gene mutation status, metastatic burden, comorbidities, nutrition status, and general condition. The second-line therapy involved an adjustment of the chemotherapy regimen and was FOLFOX (folinic acid, fluorouracil, oxaliplatin) or FOLFOXIRI. The third-line, fourth-line, or fifth-line therapy was regorafenib (Stivarga; Bayer, Leverkusen, Germany) or trifluridine plus tipiracil (Lonsurf; Taiho Pharmaceutical Co., Ltd., Tokushima, Japan). For patients with neuroendocrine carcinoma, the first-line systemic therapy was cisplatin and etoposide.

#### DEB-TACE assessment and postoperative care

All patients had an Eastern Cooperative Oncology Group score of 0–2. Systemic therapy was repeated biweekly. Serum carcinoembryonic antigen levels were measured throughout each chemotherapy cycle. Abdominal CT or MRI was performed every six cycles of therapy or if abnormal carcinoembryonic antigen levels were detected. Treatment responses were measured according to the Response Evaluation Criteria in Solid Tumors version 1.1. The treatment response was described as progressive disease, stable disease, or partial response [22]. DEB-TACE was administered when systemic therapy had failed to control the disease. Before DEB-TACE, the tumor burden of the liver was estimated according to the latest abdominal CT image. All DEB-TACE operations were performed by one team of experienced radiologists with the patient’s agreement. After each cycle of DEB-TACE, adverse events were evaluated by using the Common Terminology Criteria for Adverse Events version 4.0 [23]. A visual analog scale (VAS) was used to assess abdominal pain. VAS scores of 1–3 indicated mild (grade 1) pain, VAS scores of 4–6 indicated moderate (grade 2) pain, and VAS scores > 6 indicated severe (grade 3) pain. Laboratory data, including complete blood count and biochemistry tests, were routinely checked after each cycle of DEB-TACE. Symptomatic treatment with intravenous fluid and antipyretics was provided if fever was present after a cycle of DEB-TACE. If intra-abdominal infection was suspected, antibiotic therapy was used. Silymarin (Taiwan Biotech, Taoyuan, Taiwan) and glycyrrhizin (Stronger; Eisai, Taipei, Taiwan) were prescribed if alanine aminotransferase or aspartate aminotransferase levels were elevated. After DEB-TACE treatment, each patient maintained their scheduled systemic therapy to control the disease. Responses to DEB-TACE were evaluated by abdominal CT or MRI, which was performed as a part of systemic therapy.

### DEB-TACE

Angiography examinations were performed using Axiom Artis Zee (Siemens, Germany) to identify the hepatic arterial vasculature and to provide correlated super-selective catheterization of the tumor burden according to the CT images. DEB-TACE was performed with 100–300-μm low-compression beads (HepaSphere Microspheres and Embosphere Microspheres; Merit Medical Systems, Utah, USA). A map of the hepatic arterial vasculature was drawn, and suitable subsegmental branches for embolization were then identified. The beads were impregnated with 200–300 mg irinotecan. DEB-TACE was successful if the blood supply to the tumor was blocked or reduced. If the blood supply was not blocked or reduced, lipiodol or a gelatin sponge (Gelfoam; Pharmacia and Upjohn Company, Kalamazoo, Michigan, USA) was used to enhance the embolization. After the procedure, the main or lobar hepatic artery was checked to ensure sufficient blood flow and avoid hepatic failure. Irinotecan or epirubicin was delivered after the procedure to induce a continuous cytotoxic effect.

### Statistical analysis

Descriptive statistics are presented as proportions, medians, and means. Statistical analyses were performed using Statistical Package for the Social Sciences (version 20, International Business Machines Corporation, Armonk, NY, USA). The endpoint of follow-up was defined as the patient’s death, the last follow-up, or January 1, 2021. Overall survival (OS) was defined as the time from the date of diagnosis of mCRC to the date of death from any cause, the date of final follow-up, or the endpoint of the study. Progression-free survival (PFS) was defined as the time from the date of first DEB-TACE to the date of image findings of progressive disease. To evaluate tumor responses, CRCLM lesions on abdominal CT or angiography images were examined. Three patients had stable CRCLM that progressed to extrahepatic metastatic lesions and finally death. For these patients, the date of the last image evaluation was used to define the progressive disease response and calculate the PFS periods. Follow-up time was defined as the date of diagnosis of mCRC to the date of data collection. Subgroup analysis was performed to compare OS and PFS. Patients were divided into two groups according to when their systemic therapy had failed. Patients whose systemic therapy had failed during the first-line treatment were categorized into the first-line failure group, and patients whose systemic therapy had failed during the second-line, third-line, or fourth-line treatment were categorized into the other group. The Kaplan–Meier method was used to calculate the median OS and PFS, and a log-rank test was used to compare time-to-event distributions. A *P* value of < 0.05 indicated statistical significance.

## Results

### Patient characteristics

The median age of participants was 58 years. In total, 16 patients (45.7%) were men, and 19 patients (54.3%) were women. Most patients (34; 97.1%) had adenocarcinoma, and one patient had neuroendocrine carcinoma. Most patients (33; 94.2%) received FOLFIRI as the initial systemic chemotherapy. Targeted therapy with bevacizumab was applied for 27 patients (77.1%), and 7 patients received anti-epidermal growth factor receptor agents (6 patients [16.1%] received cetuximab and 1 patient [2.9%] received panitumumab). The patient with neuroendocrine carcinoma received cisplatin plus etoposide as systemic therapy without any targeted therapy. In total, 28 patients (80.0%) had left-sided CRC, and 7 patients (20.0%) had right-sided CRC. In total, 30 patients received an initial diagnosis of synchronous mCRC, and 5 patients had metachronous mCRC. A total of 13 patients (37.1%) had extrahepatic metastases to various organs, including the lungs, para-aortic lymph node, and peritoneum. One patient had liver, lung, and peritoneal metastases at the same time. Patient characteristics are presented in Table 1.

**Table 1:** Summary and characteristics of patients (patients, *N* = 35)

**Characteristic**	
**Age (years, median) (range)**	58 (34–80)
**Gender**	
Male	16 (45.7%)
Female	19 (54.3%)
**BMI kg/m**^**2**^** (mean) (range)**^**b**^	23.7 (17.0–33.7)
**Cancer type**	
Adenocarcinoma	34 (97.1%)
Neuroendocrine carcinoma	1 (2.9%)
**Initial systemic chemotherapy**	
FOLFIRI	33 (94.2%)
FOLFOXIRI	1 (2.9%)
Cisplatin + Etoposide	1 (2.9%)
**1st line combined target therapy**	
Bevacizumab	27 (77.1%)
Cetuximab	6 (16.1%)
Panitumumab	1 (2.9%)
None	1 (2.9%)
**Colectomy**	
Yes	20 (57.1%)
No	15 (42.9%)
**Primary tumor location**	
Right colon	7 (20.0%)
Left colon	28 (80.0%)
**Metastasis condition**	
Synchronous metastasis	30 (85.7%)
Metachronous metastasis	5 (14.3%)
**Extrahepatic metastasis**^**a**^	
Yes (%)	13 (37.1%)
Lung (% of total metastasis)	5 (14.3%)
Para-aortic lymph node (% of total metastasis)	6 (17.1%)
Peritoneum (% of total metastasis)	3 (8.6%)

^a^One patient had liver, lung, and peritoneal metastases at the same time. Thus, the sum of lung, para-aortic lymph node, and peritoneum number is fourteen

### Gene alterations

Approximately half of the patients had *RAS* wild-type genes (*KRAS* wild-type gene: 18 patients, 51.4%, *NRAS* wild-type gene: 17 patients, 48.6%), and 3 patients had the *BRAF* gene mutation (8.6%). No patients had HER2 overexpression. Gene data were unavailable for several patients. Patient gene alterations are shown in Table 2.

**Table 2:** Gene alteration status (patients, *N* = 35)

***KRAS* mutation**	
Mutation	10 (28.6%)
Wild type	18 (51.4%)
N/A	7 (20.0%)
***NRAS* mutation**	
Mutation	2 (5.7%)
Wild type	17 (48.6%)
N/A	16 (45.7%)
***BRAF* mutation**	
Mutation	3 (8.6%)
Wild type	27 (77.1%)
N/A	5 (14.3%)
**HER2 overexpression**	
Positive	0 (0%)
Negative	10 (28.6%)
N/A	25 (71.4%)
**MSI (microsatellite instability)**	
MSI-high	0 (0%)
MSI-low	21 (60.0%)
MSS (microsatellite stable)	0 (0%)
N/A	14 (40.0%)
***UGT1A1***	
TA6/TA6	13 (37.1%)
TA6/TA7	4 (11.4%)
TA7/TA7	1 (2.9%)
N/A	17 (48.6%)

### Treatment factors of DEB-TACE

**Fig. 1:**
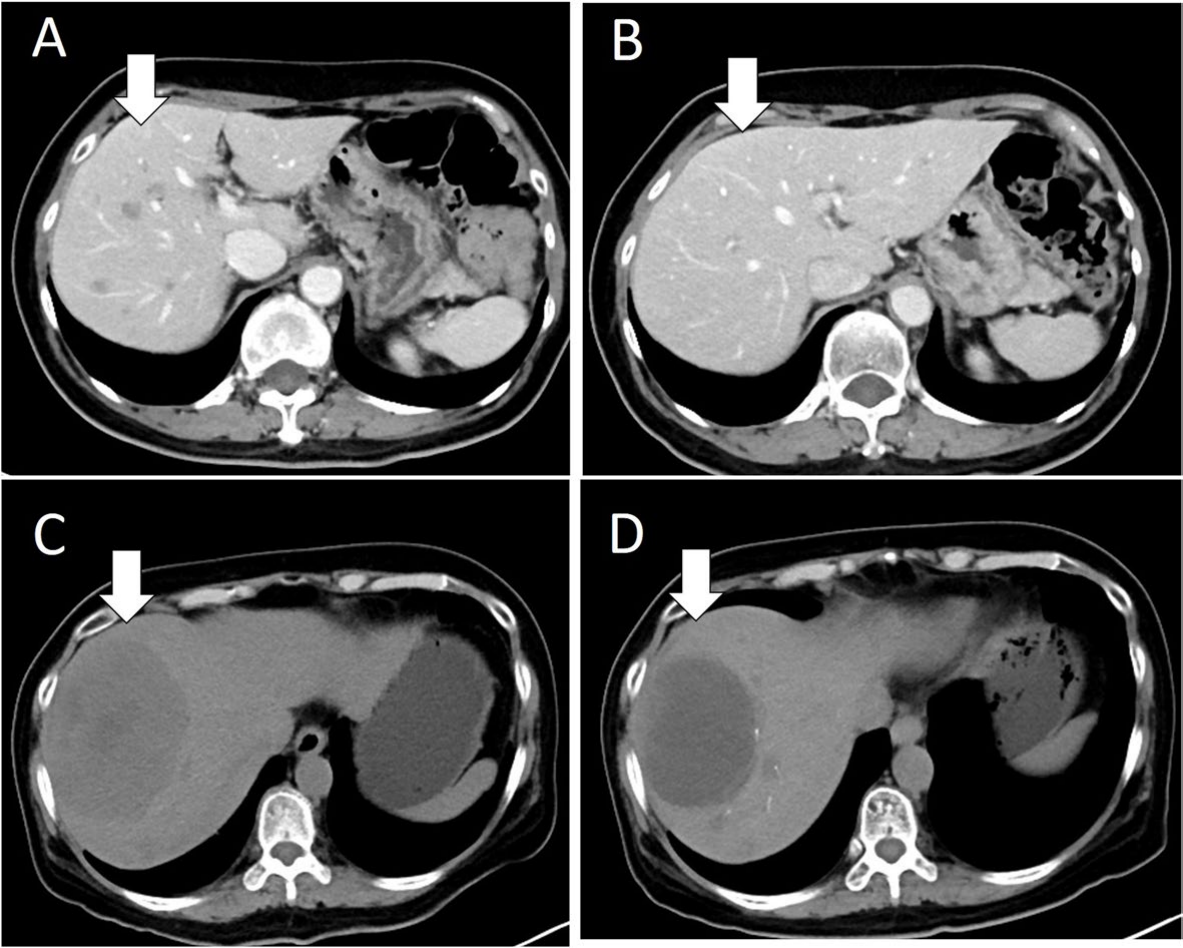
**A** Multiple CRCLM lesions were noted (arrow). The tumor burden was classified into “10 ~ 30%” but also unresectable. **B** After first DEB-TACE, tumor shrinkage was noted (arrow). **C** Large, unresectable CRCLM lesion was noted (arrow). **D** Tumor shrinkage was noted after first DEB-TACE (arrow)

TACE-DEB intervention was considered for patients whose mCRC was not effectively controlled by systemic therapy. In total, 19 patients (54.3%) started DEB-TACE after the failure of first-line systemic therapy, and 10 patients (28.5%) started DEB-TACE therapy after the failure of second-line systemic therapy. Only one patient (2.9%) received DEB-TACE intervention after the failure of fourth-line systemic therapy. Most patients (26; 74.3%) had multiple liver metastases (more than 5 lesions), and 6 patients (17.1%) had single, large, unresectable liver metastasis (Fig. 1). The liver metastasis spread to bilateral lobes in 25 patients (71.4%), and 10 metastatic tumors (28.6%) were confined to the right liver lobe. In total, 19 patients (54.3%) and 10 patients (28.6%) had tumor burdens of 10–30% and 30–50%, respectively. Six patients (17.1%) had a large tumor burden (50–70%) or hepatic vein invasion. A total of 66 DEB-TACE procedures were performed. The median number of DEB-TACE procedures was one. Three patients received five DEB-TACE procedures. In total, 90% of DEB-TACE procedures used irinotecan and 10% used epirubicin.

After the combination of systemic therapy and DEB-TACE, 5 patients (14.3%) exhibited a partial response, 13 patients (37.1%) exhibited no response that quickly turned to progressive disease, and 19 patients (54.3%) exhibited partial response + stable disease, as revealed by image findings (Fig. 1). Three patients (8.6%) did not receive any image evaluation due to disease progression and personal reasons. The treatment details of DEB-TACE are presented in Table 3.

**Table 3:** Treatment factors of transarterial chemoembolization (patients, *N* = 35) (total DEB-TACE, *N* = 66)

TACE intervention timing (*n* = 35)	
1st line therapy failure	19 (54.3%)
2nd line therapy failure	10 (28.5%)
3rd line therapy failure	5 (14.3%)
4th line therapy failure	1 (2.9%)
No. of liver metastasis (*n* = 35)	
1	6 (17.1%)
2	2 (5.7%)
4	1 (2.9%)
≧5	26 (74.3%)
Location of liver metastasis (*n* = 35)	
Right lobe	10 (28.6%)
Left lobe	0 (0%)
Both lobes	25 (71.4%)
Tumor burden of liver (*n* = 35)	
10 ~ 30%	19 (54.3%)
30 ~ 50%	10 (28.6%)
50 ~ 70%	6 (17.1%)
Number of DEB-TACE courses (*n* = 35)	
1	19 (54.3%)
2	8 (22.8%)
3	4 (11.4%)
4	1 (2.9%)
5	3 (8.6%)
Median	1
Total embolization numbers	66
DEB-TACE agent (*n* = 66)	
Irinotecan	60 (90.9%)
Epirubicin	6 (9.1%)
Best response (*n* = 35)	
Partial response (PR)	5 (14.3%)
Stable disease (SD)	14 (40.0%)
Progressive disease (PD)	13 (37.1%)
Not available^a^	3 (8.6%)
Disease control rate (PR + SD)	19 (54.3%)

^a^One patient died 2 months later after the first DEB-TACE due to intra-abdominal infection and gastrointestinal bleeding. Two patient lost follow-up after the first DEB-TACE

### Adverse events of DEB-TACE

Abdominal pain was the most common adverse event of DEB-TACE. Abdominal pain was observed in 77.3% of a total of 66 procedures, of which 19.7% exhibited grade 3 adverse events. Abdominal pain subsided 2–5 days after symptomatic treatment. Nausea and vomiting were observed in 28.8% and 21.2% of DEB-TACE procedures, respectively, but were assessed as grade 1 only and subsided after rest or symptomatic treatment. Grade 1 fever was observed in 21.2% of DEB-TACE procedures and was treated with antipyretics. Grade 2 fever was observed in 1.5% of DEB-TACE procedures. No patient had a grade 3 fever. Elevated alanine aminotransferase and aspartate aminotransferase levels were observed in 75.8% and 95.5% of DEB-TACE procedures, respectively, and grade 3 elevations of alanine aminotransferase and aspartate aminotransferase were observed in 12.1% and 33.3% of DEB-TACE procedures, respectively. These elevations were subsequently corrected by conservative treatment. Anemia was detected in 50% of DEB-TACE procedures, but most of these anemia episodes were grades 1 or 2. No incidence of neutropenia after DEB-TACE procedures occurred. Among the 66 DEB-TACE procedures, four episodes (6.1%) of grade 3 intra-abdominal infection were noted in four patients. Leukocytosis with a white blood cell count of > 20,000/µL and C-reactive protein > 200 mg/L were observed in these four cases, which were accompanied by abdominal pain with or without low-grade fever. All patients improved uneventfully after antibiotic therapy for 7–10 days without surgical intervention. No evidence of cholecystitis was observed in any of the patients. Adverse events are listed in Table 4.

**Table 4:** Common terminology criteria for adverse event (CTCAE) adverse events of transarterial chemoembolization (*n* = 66)

Abdominal pain	51 (77.3%)
Grade 1	23 (34.8%)
Grade 2	15 (22.3)
Grade 3	13 (19.7%)
Nausea	19 (28.8%)
Grade 1	19 (28.8%)
Grade 2	0 (0%)
Grade 3	0 (0%)
Vomiting	14 (21.2%)
Grade 1	14 (21.2%)
Grade 2	0 (0%)
Grade 3	0 (0%)
Fever	15 (22.3%)
Grade 1	14 (21.2%)
Grade 2	1 (1.5%)
Grade 3	0 (0%)
Alanine aminotransferase increased	50 (75.8%)
Grade 1	33 (50.0%)
Grade 2	9 (13.6%)
Grade 3	8 (12.1%)
Aspartate aminotransferase increased	63 (95.5%)
Grade 1	21 (31.8%)
Grade 2	20 (30.3%)
Grade 3	22 (33.3%)
Anemia	33 (50%)
Grade 1	20 (30.3%)
Grade 2	11 (16.7%)
Grade 3	2 (3.0%)
Neutropenia	0 (0%)
Intra-abdominal infection	4 (6.1%)
Grade 1	0 (0%)
Grade 2	0 (0%)
Grade 3	4 (6.1%)
Cholecystitis	0 (0%)

### Survival and treatment outcome

**Fig. 2:**
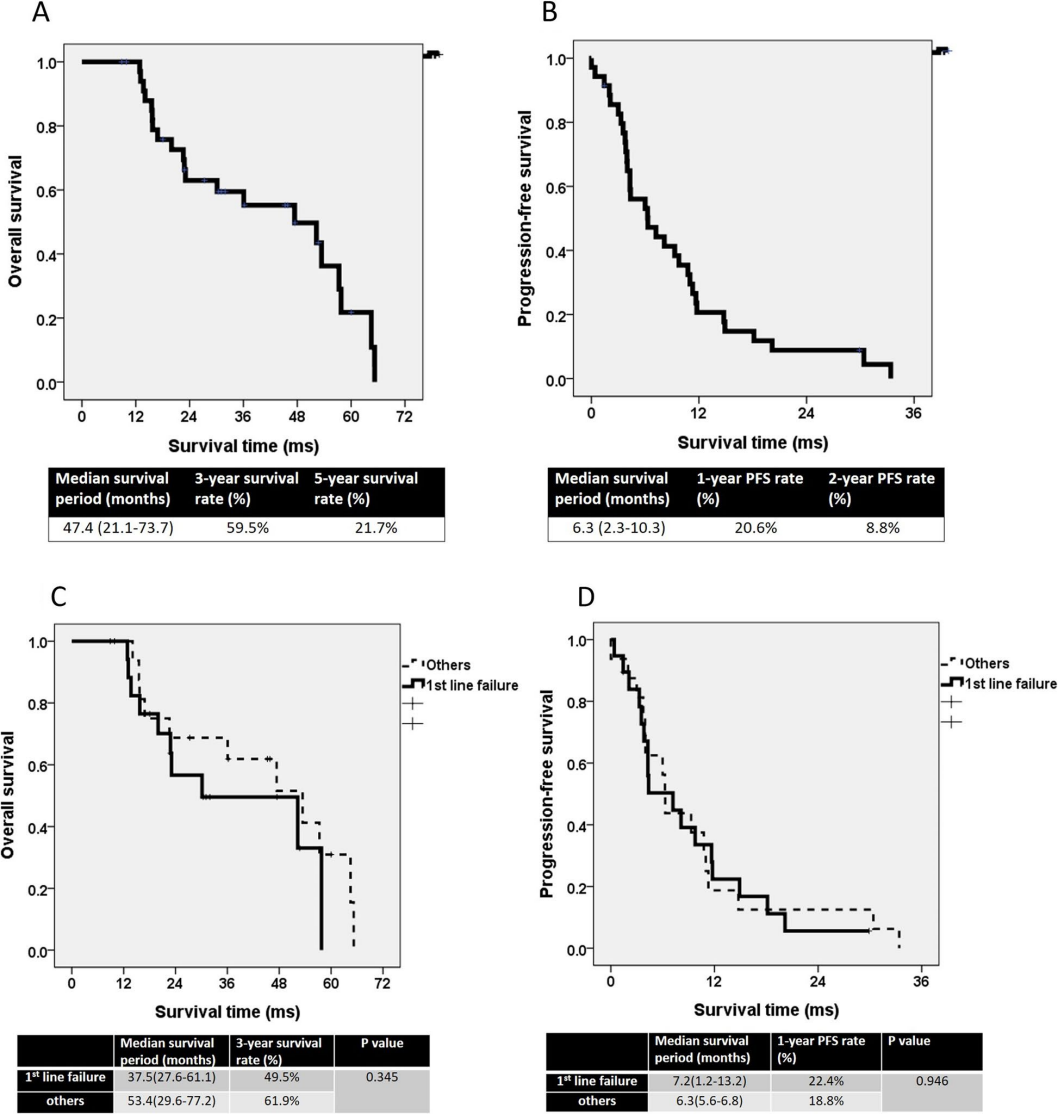
**A** The overall survival curve. **B** The progression-free survival curve. **C** The overall survival curve of “1st line failure” and “others” groups. **D** The progression-free curve of “1st line failure” and “others” groups

The median follow-up period was 43.7 months (range: 9.9–97.9 months). The estimated median OS period was 47.4 months (range: 21.1–73.7 months). The estimated 3-year and 5-year OS rates were 59.5% and 21.7%, respectively (Fig. 2A). The estimated median PFS period was 6.3 months (range: 2.3–10.3 months). The 1-year and 2-year PFS rates were 20.6% and 8.8%, respectively (Fig. 2B). The estimated median OS period in the first-line failure group was 37.5 months (range: 27.6–61.1 months) and in the other group was 53.4 months (range: 29.6–77.2 months). The estimated 3-year OS rates in the first-line failure and other groups were 49.5% and 61.9%, respectively. No significant differences in OS were observed between the two groups (*P* = 0.345). The estimated PFS period in the first-line failure group was 7.2 months (range: 1.2–13.2 months) and in the other group was 6.3 months (range: 5.6–6.8 months). The estimated 1-year PFS rates in the first-line failure and other groups were 22.4% and 18.8%, respectively. The difference in PFS between the two groups was nonsignificant (*P* = 0.946).

## Discussion

CRCLM accounts for nearly half of mCRC. Several treatment methods are available for CRCLM [6, 12]. Systemic therapy has been established as the standard initial treatment for CRCLM, and surgical resection of liver metastases has shown potential for curing the disease [24, 25]; however, 75–80% of metastatic lesions associated with CRCLM are deemed unresectable [17, 26]. In such cases, chemotherapy with FOLFIRI or FOLFOX in combination with targeted therapy with specific drugs, such as cetuximab, is recommended. This approach can yield an overall response rate of 44–72% [27]. Nevertheless, 65–75% of patients with CRCLM do not receive curative surgery [24, 25, 27]. In total, 10–27% of patients who receive chemotherapy experience grade 3 or grade 4 adverse events, such as neutropenia and skin toxicity [24, 25]. When patients with CRCLM exhibit no response to systemic therapy, second-line or third-line therapy is necessary to control the disease; however, unfavorable treatment outcomes are anticipated. In previous studies, the median PFS period for patients who received second-line therapy was approximately 5.2–7.7 months, with a corresponding median OS of approximately 11.6–14.6 months [28, 29].

Conventional TACE (cTACE) is a combination of chemotherapy and embolization agents. cTACE has cytotoxic and ischemic effects and has been widely used for hepatocellular carcinoma [13, 30]. cTACE has also been used to control CRCLM. With embolization caused by lipiodol and arterial infusion of mitomycin C, irinotecan, and cisplatin, cTACE is effective at controlling unresectable CRCLM. And median OS and PFS periods in unresectable CRCLM patients who received cTACE were 25.8 and 10.8 months, respectively [30, 31]. DEB-TACE provides the continuous intra-arterial release of chemotherapy agents by using permanent microspheres [15, 26]. In patients with CRCLM who have failed to respond to chemotherapy, DEB-TACE can improve their survival [3]. DEB-TACE with irinotecan yielded a longer OS period than did systemic irinotecan therapy (22 vs. 15 months) [32]. However, few studies have evaluated the combination of DEB-TACE and systemic therapy with chemotherapy and targeted therapy. Stutz et al. examined 27 patients with CRCLM at a single institute who failed to respond to systemic therapy with FOLFOX, FOLFIRI, or FOLFOXIRI and targeted therapy consisting of bevacizumab and cetuximab [33]. The study concluded that DEB-TACE is an effective treatment for CRCLM; however, the OS period in that study was only 5.4 months; therefore, the results are inconclusive [30, 33]. The present study provides more evidence about the role of DEB-TACE in the treatment of CRCLM. Standard treatment with systemic chemotherapy and targeted therapy yielded a median OS period of 47.4 months and 3-year and 5-year OS rates of 59.5% and 21.7%, respectively. These results suggest that DEB-TACE is an acceptable treatment modality for CRCLM.

A systematic review of 13 studies with 850 patients evaluated treatment outcomes in patients with unresectable CRCLM receiving DEB-TACE [34]. In the review, the average OS period was 16.8 months, and the PFS period was 8.1 months. Compared with that review, the PFS period was similar (6.3 months) but the OS period was longer (47.4 months) in the present study. A possible reason for the longer OS period in the present study is that patients in the present study received combined targeted therapy. After the introduction of anti-vascular endothelial growth factor and anti-epidermal growth factor receptor agents in 2010–2015, the survival rates of patients with mCRC improved [35]. Even with metastasis progression, mCRC lesions can be controlled by using advanced targeted therapy agents, thereby extending the patient’s survival period. In our previous study, the median OS period of patients with mCRC who received standard systemic therapy was 30–40 months [19]. In the present study, the median OS period was 47.4 months, and the favorable oncological outcomes suggest that DEB-TACE intervention did not delay or replace standard systemic therapy.

Resection of metastatic lesions is a key factor for improving survival outcomes in patients with mCRC receiving systemic therapy [3, 6]. DEB-TACE can further shrink liver metastases associated with CRC and increase the likelihood of successful metastasectomy [8]. In a recent study that involved 42 patients with unresectable liver metastases who were treated with DEB-TACE, a 100% DCR and 19% complete response rate were observed [36]. DEB-TACE alone was demonstrated to convert potentially resectable liver metastases to resectable, and a pathology report revealed a 77.3% tumor pathologic response [37]. In our previous study, the median OS period of patients with mCRC who received systemic therapy plus metastasectomy was 48 months [38]. In the present study, although none of the liver metastases became resectable after treatment, the median OS period was similar to that in our previous study (47.4 months), and DCR was 54.3%. Furthermore, in the present study, 37.1% of the patients had extrahepatic metastases to the lung, peritoneum, or para-aortic lymph node. This finding suggests that DEB-TACE provides effective local disease control and potential survival benefits to mCRC patients with unresectable liver metastases. Even with extrahepatic CRC metastasis, the combination of DEB-TACE and systemic therapy can result in a favorable DCR and extend the survival period.

Palliative therapy that included DEB-TACE was demonstrated to provide survival benefits [30]. DEB-TACE is an effective treatment for CRCLM [8, 32, 39]. A consensus has not been reached regarding the optimal timing of DEB-TACE during treatment for mCRC. Martin et al. analyzed the data of 55 patients with CRCLM who underwent 99 TACE interventions after the failure of systemic therapy [12]. The study concluded that TACE intervention is beneficial for patients with CRCLM who failed to respond to first-line and second-line therapy. A multicenter phase-2 study examined 57 patients with CRCLM who underwent DEB-TACE and modified FOLFOX6 therapy [39]. Although the study did not conclude that DEB-TACE was an effective front-line therapy, the DCR and OS outcomes were promising. The median OS period was 37.4 months, and the median PFS period was 10.8 months [39]. In the present study, the median PFS period for patients who received DEB-TACE after the failure of first-line therapy was 7.2 months and for patients who received DEB-TACE after the failure of second-line therapy was 6.3 months (Fig. 2D). Although early intervention (i.e., after the failure of first-line therapy) corresponded to a longer PFS period, no significant difference in OS was observed between the first-line therapy failure and other groups.

The results for the difference in OS between the first-line failure group and the other group contrasted with those for the difference in PFS between the two groups. The median OS period in the other group was 53.4 months, which was longer than the OS period in the first-line failure group (37.5 months). Although the difference was nonsignificant (*P* = 0.345), this finding was confusing. We believe that this finding may be due to the limited sample size. The presence of extrahepatic metastasis was a risk factor for poor prognosis [12, 30]. The first-line failure group contained 19 patients, but 8 patients (42.1%) had extrahepatic metastases. The other group had 16 patients, and only 5 patients (31.3%) had extrahepatic metastases. The shorter OS period in the first-line failure group may be due to the higher proportion of extrahepatic metastases. Even though DEB-TACE can effectively control CRCLM, the primary treatment for mCRC is systemic therapy [28, 30].

DEB-TACE provides a continuous and highly concentrated dose of chemotherapy agents and does not cause systemic adverse events [10]. Elevation of liver enzymes is the most common adverse event, occurring in 75.8–95.5% of procedures in the present study. Abdominal pain was observed in 77.3% of procedures. Abdominal pain, nausea, fever, and liver dysfunction have been reported in various studies [8, 36, 40]. Nevertheless, grade 3 and grade 4 adverse events are rare and subside after conservative treatment [8, 36, 40]. In the present study, four episodes of grade 3 intra-abdominal infection were observed in four patients. These patients experienced fever, abdominal pain, elevated C-reactive protein levels, and leukocytosis 1–3 days after DEB-TACE. Supportive treatment with intravenous fluid and antibiotic therapy was indicated. These patients recovered 10–14 days after hospitalization. No TACE-related mortality was noted. In our opinion, close observation with routine blood biochemistry testing is warranted after each DEB-TACE intervention.

The analysis of data from only 35 patients is the major limitation of this article. This single-center retrospective study had a small sample size and few DEB-TACE treatment cycles. Therefore, subgroup analysis was not feasible. Paradoxical results were observed for the results for the differences in OS and PFS between the first-line failure and other groups. OS and PFS between the first-line failure and other groups should be compared. Various data (i.e., gene alteration status) were not available. For example, *KRAS* and *NRAS* gene mutation status were unavailable for 20.0% and 45.7% of patients, respectively. Due to missing data, we failed to identify a relationship between the gene type and health outcomes. Moreover, heterogeneity was noted in this study. Cancer type, chemotherapy, targeted therapy, and DEB-TACE agents were not consistent due to the retrospective design of this study. These characteristics impeded our analysis. Further prospective trials are warranted to investigate the role of DEB-TACE in the treatment of CRCLM.

## Conclusion

The present study described the clinical outcomes of the combination of DEB-TACE, chemotherapy, and targeted therapy for CRCLM. DEB-TACE can lead to an adequate DCR and favorable survival outcomes in patients with mCRC. Early intervention with DEB-TACE, especially following first-line therapy failure, provides optimal control of CRCLM. Severe adverse reactions to DEB-TACE are rare. Mild and moderate adverse reactions to DEB-TACE are manageable. Further prospective, randomized controlled trials are warranted to clarify the role of combined DEB-TACE and chemotherapy/targeted therapy in the treatment of CRCLM.

## Data Availability

The data and materials contributing to this article may be made available upon request by sending an e-mail to the corresponding author.

## Abbreviations

CRC: Colorectal cancer
mCRC: Metastatic colorectal cancer
CRCLM: Colorectal cancer with liver metastasis
CT: Computed tomography
DEBs: Drug-eluting beads
DCR: Disease control rate
DEB-TACE: Transarterial chemoembolization with drug-eluting beads
FOLFIRI: Folinic acid, 5-fluorouracil, and irinotecan
FOLFOX: Folinic acid, fluorouracil, and oxaliplatin
FOLFOXIRI: Folinic acid, 5-fluorouracil, oxaliplatin, and irinotecan
MRI: Magnetic resonance imaging
OS: Overall survival
PFS: Progression-free survival
TACE: Transarterial chemoembolization
cTACE: Conventional transarterial chemoembolization
VAS: Visual analogue scale
